# Is a Cancer Diagnosis Associated with Subsequent Risk of Transient Global Amnesia?

**DOI:** 10.1371/journal.pone.0122960

**Published:** 2015-04-07

**Authors:** Jianwei Zhu, Donghao Lu, Olafur Sveinsson, Karin Wirdefeldt, Katja Fall, Fredrik Piehl, Unnur Valdimarsdóttir, Fang Fang

**Affiliations:** 1 Department of Medical Epidemiology and Biostatistics, Karolinska Institutet, Stockholm, Sweden; 2 Department of Neurology, Karolinska University Hospital, Stockholm, Sweden; 3 Department of Clinical Neuroscience, Karolinska Institutet, Stockholm, Sweden; 4 Clinical Epidemiology and Biostatistics Unit, Örebro University, Örebro, Sweden; 5 Center of Public Health, University of Iceland, Reykjavík, Iceland; 6 Department of Orthopedics, Shandong Provincial Hospital Affiliated to Shandong University, Jinan, China; Sudbury Regional Hospital, CANADA

## Abstract

**Background:**

Psychological stress has been associated with transient global amnesia (TGA). Whether a cancer diagnosis, a severely stressful life event, is associated with subsequent risk of TGA has not been studied.

**Methods:**

Based on the Swedish Cancer Register and Patient Register, we conducted a prospective cohort study including 5,365,608 Swedes at age 30 and above during 2001–2009 to examine the relative risk of TGA among cancer patients, as compared to cancer-free individuals. Incidence rate ratios (IRRs) and their 95% confidence intervals (CIs) derived from Poisson regression were used as estimates of the association between cancer diagnosis and the risk of TGA.

**Results:**

During the study 322,558 individuals (6.01%) received a first diagnosis of cancer. We identified 210 cases of TGA among the cancer patients (incidence rate, 0.22 per 1000 person-years) and 4,887 TGA cases among the cancer-free individuals (incidence rate, 0.12 per 1000 person-years). Overall, after adjustment for age, sex, calendar year, socioeconomic status, education and civil status, cancer patients had no increased risk of TGA than the cancer-free individuals (IRR, 0.99; 95% CI, 0.86–1.13). The IRRs did not differ over time since cancer diagnosis or across individual cancer types. The null association was neither modified by sex, calendar period or age.

**Conclusion:**

Our study did not provide support for the hypothesis that patients with a new diagnosis of cancer display a higher risk of TGA than cancer-free individuals.

## Introduction

Transient global amnesia (TGA) is one of the most striking syndromes in clinical neurology, characterized by a sudden onset of profound global amnesia (both retrograde and anterograde) which usually lasts less than 24 hours and typically resolves within a few hours [1]. TGA occurs most common at the age of 50–80 years [2] and has an overall incidence of 5–11 per 100,000 person-years [3–5]. The incidence is largely age dependent; and among middle aged and older individuals, the incidence has been estimated to reach 30 cases per 100,000 person-years [6, 7]. Given a lack of diagnostic tests, TGA remains a clinical diagnosis where other causes of amnesia such as epilepsy or stroke need to be excluded [7].

Although little is known regarding the etiology of TGA [1, 3, 8–11], empirical evidence suggests a higher than expected prevalence of precipitating events, including both emotionally and physically stressful situations before the onset of TGA episodes [6, 7, 9, 12, 13]. Other studies have also suggested that TGA patients are more likely to carry a psychopathological personality or demonstrate an emotional instability [2]. The potential triggering events for TGA have further proposed to induce a strong stress response, leading to subsequent changes of homeostasis [2, 8, 14, 15]. More recently, Döhring *et al*. [15] found that TGA patients cope with stress less efficiently and exhibit an elevated anxiety level. The underlying mechanism for the proposed stress-TGA link has also been explored recently. For example, psychological disturbance may cause transient alteration in brain metabolism and, subsequently, amnesia [16]. Emotional stress may also result in hippocampal dysfunction, leading to a consequent loss of memory [12]. Furthermore, Evans *et al*. [17] hypothesized that the amnesic syndrome of TGA may be triggered by a temporary shutdown of neurotransmitters during a depression-like attack. On the contrary, no epidemiological study has to our knowledge examined the association of severely stressful life events with the risk of TGA.

The idea that a cancer diagnosis, independent of the cancer disease itself or the treatment, may serve as a severe psychological stress and lead to serious health consequences has only recently been developed [18–20]. Being diagnosed and living with cancer is highly stressful. Psychological distress likely persists during the entire disease course, including the diagnostic workup, primary cancer treatment, disease recurrence, metastasis, and the eventual end of life stage. Among others, we have previously reported highly increased risks of cardiovascular diseases and suicide immediately after cancer diagnosis [21–24]. However, suicide and cardiovascular diseases likely represent only the tip of iceberg for the enormous psychological distress a newly diagnosed cancer patient experiences. To this end, we hypothesized that a cancer diagnosis might serve as a potential emotional trigger for TGA.

## Materials and Methods

### Study design

We conducted a prospective cohort study based on the Swedish Population and Housing Census in 1990, including 5,365,608 individuals born in Sweden. All participants were free of cancer while entering to the cohort and 30 years of age or older between January 1st 2001 and December 31st 2009. Information regarding participants’ characteristics, including sex, date of birth, civil status, and socioeconomic status, was collected from this Census. Using the individually unique personal identification numbers, the study cohort was linked to the nationwide Cancer, Causes of Death, Migration, Education, and Patient Registers. Follow-up of the study participants started from January 1st 2001 or their 30th birthday, whichever came later, until the date of first TGA diagnosis, death, emigration, or December 31st 2009, whichever occurred first. The cancer-free individuals contributed all accumulated person-time to the unexposed group; while the cancer patients contributed person-time to the unexposed group before the date of diagnosis and to the exposed group afterwards.

### Ascertainment of cancer

In Sweden, clinicians and pathologists have been required by law to report all newly diagnosed cancer cases to the Swedish Cancer Register since 1958; and the completeness of this register approaches 100% [25]. In total, we identified 326,739 individuals who received their first cancer diagnosis during follow-up. Since we aimed to study the risk of TGA among cancer patients, individuals with a cancer diagnosed through autopsy were excluded (N = 4,181), leaving 322,558 in the analysis. Among these cancer patients, the most common cancer types were prostate cancer (N = 69,373), breast cancer (N = 45,238), colorectal cancer (N = 36,777), and melanoma or other skin cancers (N = 32,988).

### Ascertainment of TGA

TGA was identified from the Swedish Patient Register, defined as an inpatient or outpatient hospital visit with TGA as the main discharge diagnosis. As described previously, the Patient Register started to collect hospital discharge records in 1964/1965 and has a nationwide coverage for hospital discharge records since 1987 [26]. Outpatient visits for hospital-based specialist care, but not general practitioner care, have also been recorded in this register since 2001. This outpatient visit registration is estimated to cover >80% of the entire Sweden [26]. In the Patient Register, all discharge diagnoses are coded according to the Swedish revision of the 10th International Classifications of Diseases (ICD) codes since 1997. We used G454 as the ICD-10 code for TGA.

### Validation of hospital-based TGA diagnosis

The discharge diagnoses of the Swedish Patient Register, including many neurological disorders, have been widely validated, with a generally high positive predictive value (85%–95%). To assess the accuracy of hospital-based TGA diagnosis, we thoroughly reviewed the medical records of all patients receiving a diagnosis of TGA in two different hospitals in Sweden during 2006–2009, including Karolinska University Hospital (a large university hospital; N = 159) and Nyköping Hospital (a smaller provincial hospital; N = 38). The diagnosis of TGA was verified if the following diagnostic criteria by Hodges [3] were fulfilled: (i) presence of an anterograde amnesia that is witnessed by an observer, (ii) no clouding of consciousness or loss of personal identity, (iii) cognitive impairment limited to amnesia, (iv) no focal neurological or epileptic signs, (v) no recent history of head trauma or seizures, and (vi) resolution of symptoms within 24 hours. Two neurologists independently reviewed the medical records first and made joint decisions by consensus. Among all the 197 cases, 185 (94%; 95% confidence interval: 91%-97%) fulfilled the criteria, while 12 (6%; 95% confidence interval: 3%-9%) were classified as not TGA. The accuracy of TGA diagnosis did not appear to differ largely between hospitals. In Karolinska University Hospital, 148 of the 159 TGA cases (93%; 95% confidence interval: 89%-97%), and in Nyköping Hospital, 37 of the 38 TGA cases (97%; 95% confidence interval: 92%-102%), fulfilled these criteria.

### Statistical analysis

We first calculated the crude incidence rates of TGA through dividing the observed numbers of TGA cases by the accumulated person-years among individuals with and without diagnosed cancer. As a measure of the association between a cancer diagnosis and the risk of TGA, we then calculated the incidence rate ratios (IRRs) and their 95% confidence intervals (CIs) based on Poisson regression modeling. IRR was calculated as the ratio of TGA incidence rate of the cancer patients to that of the cancer-free participants. In all statistical models, we adjusted for age at follow-up (≤59, 60–64, 65–69, 70–74, 75–79, or ≥80 years), sex, calendar year of follow-up, civil status (cohabitating or not-cohabitating), socioeconomic status (blue-collar, white-collar, self-employed, or unclassified) and education levels (≥9 years, <9 years, or missing). Information on the highest obtained educational level was ascertained from linking the study cohort to the Swedish Education Register.

To assess potential effect modifiers of the studied association, we further stratified the analyses by sex, age at follow-up and calendar period of follow-up (2001–2003, 2004–2006, and 2007–2009). Furthermore, IRRs were separately calculated according to time since cancer diagnosis (first, second, third year after diagnosis, and beyond) and for individual cancer types (prostate cancer, breast cancer, colorectal cancer, and skin cancer).

TGA has a recurrence rate of 6–10% [2], and the impact of cancer diagnosis on risk of TGA may differ between individuals with and without a previous episode of TGA. Previous history of TGA was therefore ascertained from the Swedish Patient Register; since TGA could only be identified through the ICD-10 code, we ascertained previous history of TGA only from 1997 onward. Individuals were classified as having previous TGA during the entire follow-up if they were diagnosed with TGA at least once before entry to the cohort. Cancer patients were classified as having previous TGA if they had at least one TGA episode before cancer diagnosis.

All statistical analyses were carried out using SAS 9.3 software (SAS Institute, North Carolina, United States). The study was approved by the Regional Ethics Vetting Board at the Karolinska Institutet. All the individual records were anonymized and de-identified prior to analysis.

## Results

The baseline characteristics of the study participants are shown in Table 1. During follow-up, 4,887 TGA cases were observed among the cancer-free individuals (incidence rate, 0.12 per 1000 person-years), while 210 TGA cases were identified among the cancer patients (incidence rate, 0.22 per 1000 person-years). There were more men who developed TGA among the cancer patients (57.14%), but slightly more women among the cancer-free group (51.07%). Cancer patients who developed TGA later on were less well educated as compared to other cancer patients; similar difference was however not observed among the cancer-free group. For both cancer patients and cancer-free individuals, individuals with TGA were more likely to be non-cohabitating and white-collar workers than individuals without TGA (Table 1).

**Table 1:** Baseline characteristics of study participants in a cohort study of cancer diagnosis and transient global amnesia (TGA) in Sweden, 2001–2009.

**Characteristics**	**Cancer patients**		**Cancer-free individuals**	
	**Total**	**TGA**	**Total**	**TGA**
**N**	322,558	210	5,365,608	4,887
**Mean age at entry, years (SD)**	68.13 (12.82)	66.59 (7.60)	50.16 (17.12)	59.72 (9.36)
**Mean follow-up, years (SD)**	3.01 (2.54)	2.58 (2.04)	7.62 (2.52)	5.02 (2.61)
**Sex, N (%)**				
Male	171,183 (53.07)	120 (57.14)	2,652,896 (49.44)	2,391 (48.93)
Female	151,375 (46.93)	90 (42.86)	2,712,701 (50.56)	2,496 (51.07)
**Educational level, N (%)**				
≥9 years	183,508 (56.89)	151 (71.90)	3,822,871 (71.25)	3,373 (69.02)
<9 years	135,321 (41.95)	59 (28.10)	1,467,569 (27.35)	1,506 (30.82)
Missing	3,729 (1.16)	0 (0)	75,157 (1.40)	8 (0.16)
**Civil status, N (%)**				
Non-cohabitating	214,719 (66.57)	164 (78.10)	2,413,856 (44.99)	3,754 (76.82)
Cohabitating	107,839 (33.43)	46 (21.90)	2,951,741 (55.01)	1,133 (23.18)
**Previous TGA, N (%)**	372 (0.12)	2 (0.95)	1,107 (0.02)	63 (1.29)
**Socioeconomic status, N (%)**				
Blue-collar	117,869 (36.54)	55 (26.19)	1,936,130 (36.08)	1,553 (31.78)
White-collar	133,053 (41.25)	133 (63.33)	1,742,667 (32.48)	2,657 (54.37)
Self-employed	26,919 (8.35)	18 (8.57)	276,920 (5.16)	414 (8.47)
Unclassified	44,717 (13.86)	4 (1.90)	1,409,880 (26.28)	263 (5.38)

After multivariable adjustments, no overall association was observed between a cancer diagnosis and the risk of TGA (IRR, 0.99; 95% CI, 0.86–1.13) (Table 2). The association did not appear to differ largely between men and women; neither did it differ by age at or calendar period of follow-up (Table 2). A statistically significant association was observed for the age group of 65–69 years (IRR, 1.31; 95% CI, 1.02–1.66); this age group had also the highest incidence rates of TGA both among cancer patients and cancer-free individuals. The incidence rate of TGA was higher among individuals with preexisting TGA, regardless of cancer diagnosis. However, the IRRs of TGA after a cancer diagnosis did not differ by the status of previous TGA (Table 2).

**Table 2:** The crude incidence rates (IRs, per 1000 person-years) and incidence rate ratios (IRRs) of transient global amnesia (TGA) after cancer diagnosis, according to sex, calendar period of follow-up, age at follow-up, and previous TGA, a cohort study in Sweden, 2001–2009.

	**Cancer patients**		**Cancer-free individuals**		**IRR (95% CI)** ^a^
	**N**	**IR**	**N**	**IR**	
**Overall**	210	0.22	4,887	0.12	0.99 (0.86–1.13)
**Calendar period of follow-up**					
2001–2003	22	0.18	1,254	0.09	1.20 (0.76–1.78)
2004–2006	64	0.19	1,586	0.12	1.02 (0.78–1.30)
2007–2009	124	0.24	2,047	0.15	0.95 (0.79–1.13)
**Sex**					
Male	120	0.24	2,391	0.12	1.04 (0.85–1.25)
Female	90	0.19	2,496	0.12	0.96 (0.78–1.18)
**Age at follow-up, years**					
30–59	14	0.06	1,308	0.05	0.93 (0.52–1.50)
60–64	41	0.31	1,123	0.29	1.00 (0.72–1.34)
65–69	70	0.50	1,010	0.35	1.31 (1.02–1.66)
70–74	35	0.25	757	0.32	0.72 (0.50–1.00)
75–79	28	0.21	440	0.21	0.90 (0.60–1.30)
≥80	22	0.11	249	0.08	1.13 (0.71–1.72)
**Previous TGA**					
Yes	2	1.95	63	7.44	0.33 (0.05–1.09)
No	208	0.21	4,824	0.12	0.99 (0.86–1.14)
**Educational level**					
≥9 years	151	0.26	3,373	0.11	1.04 (0.88–1.22)
<9 years	59	0.16	1,514	0.14	0.87 (0.66–1.12)
**Civil status**					
Not-cohabitating	164	0.25	3,754	0.19	0.93 (0.79–1.08)
Cohabitating	46	0.15	1,133	0.05	1.24 (0.91–1.66)
**Socioeconomic status**					
Blue collar	55	0.16	1,553	0.10	0.90 (0.68–1.16)
White collar	133	0.31	2,657	0.18	1.08 (0.90–1.29)
Self-employed	18	0.22	414	0.19	0.88 (0.53–1.38)
Unclassified	4	0.04	263	0.03	0.45 (0.14–1.07)

^a^ IRRs were adjusted for age at follow-up (≤59, 60–64, 65–69, 70–74, 75–79, and ≥80 years), sex, calendar period of follow-up (3-year groups), civil status (cohabitating or non-cohabitating), socioeconomic status (blue-collar, white-collar, self-employed, or unclassified), and educational level (≥9 years, <9 years, or missing); CI: confidence interval.

The null association between cancer diagnosis and TGA was furthermore consistent across different time periods after cancer diagnosis and did not appear to differ largely across the main cancer types (Table 3).

**Table 3:** The incidence rate ratios (IRRs) of transient global amnesia after cancer diagnosis according to time since cancer diagnosis and by different cancer types, a cohort study in Sweden, 2001–2009.

	**Any cancer**		**Prostate cancer**		**Breast cancer**		**Colon cancer**		**Skin cancer**	
	**N**	**IRR (95% CI)** ^a^	**N**	**IRR (95% CI)** ^a^	**N**	**IRR (95% CI)** ^a^	**N**	**IRR (95% CI)** ^a^	**N**	**IRR (95% CI)** ^a^
**Cancer-free individuals**	4,887	1.0	4,887	1.0	4,887	1.0	4,887	1.0	4,887	1.0
**After cancer diagnosis**	210	0.99 (0.86–1.13)	75	1.12 (0.88–1.40)	33	0.93 (0.65–1.29)	28	1.25 (0.85–1.79)	20	0.94 (0.59–1.42)
1st year	56	1.06 (0.80–1.36)	17	1.09 (0.65–1.70)	12	1.68 (0.90–2.82)	6	0.99 (0.39–2.01)	2	0.39 (0.06–1.20)
2nd year	42	1.01 (0.73–1.35)	16	1.20 (0.70–1.89)	2	0.31 (0.05–0.97)	6	1.30 (0.52–2.63)	3	0.71 (0.18–1.83)
3rd year	39	1.14 (0.82–1.54)	16	1.40 (0.82–2.21)	7	1.24 (0.53–2.40)	3	0.84 (0.21–2.17)	6	1.73 (0.69–3.51)
>3 years	73	0.87 (0.68–1.09)	26	0.97 (0.64–1.39)	12	0.73 (0.39–1.23)	13	1.62 (0.89–2.68)	9	1.07 (0.51–1.93)

^a^ IRRs were adjusted for age at follow-up (≤59, 60–64, 65–69, 70–74, 75–79, and ≥80 years), sex, calendar period of follow-up (3-year groups), civil status (cohabitating or non-cohabitating), socioeconomic status (blue-collar, white-collar, self-employed, or unclassified), and educational level (≥9 years, <9 years, or missing); CI: confidence interval

## Discussion

Based on a cohort of more than five million individuals, we found no different risk for TGA after cancer diagnosis as compared to the cancer-free general population. The null association was not modified by age, sex, or calendar period and did not differ for different time periods after cancer diagnosis or different cancer types.

Physically and emotionally stressful events have both been proposed to precipitate TGA [27]. Based on a comprehensive literature review and scrutinization of 142 TGA cases, Quinette *et al*. reported that 28–29% of the patients had emotional stress and 25–31% had physical effort as the close precipitating events before the onset of a TGA episode [2]. When compared to gender- and age-matched controls, emotional stress was however not more prevalent among TGA cases and therefore not associated with the risk of TGA [2]. Using patients with transient ischemic attack (TIA) as controls, Inzitari *et al*. [13] did not either find significantly different prevalence of stressful life events (e.g., sudden death of a close relative) during the year preceding TGA and TIA. In line with these null findings, our study did not provide any evidence for a causal relationship between a cancer diagnosis—a severely stressful life event—and the subsequent risk of TGA. These results could however not be used to argue against the possibility that other kinds of stressful life events may still be causally related to TGA, either as a trigger or chronic cause.

To the best of our knowledge, this is the first report addressing TGA occurrences among cancer patients. After a diagnosis of cancer, patients may persistently suffer from severe psychological stress [28], while waiting for the diagnosis [29] and making treatment decisions [30], or due to treatment side effects [31], increasing physical distress [32], disease recurrence and metastasis [33], and eventually the pending death [33]. Previous studies have reported increased risks of different severe health outcomes including suicide [34, 35] and cardiovascular diseases [36] among cancer patients from immediately after diagnosis to the end of life, reflecting severe stress experienced by the patients during different stages of cancer. The different findings between these outcomes and TGA may be informative on the potentially differing mechanisms underlying TGA and other outcomes sensitive to strong stressors.

Strengths of our study include the large-scale population-based cohort design, the complete follow-up, and the independently and prospectively collected data on cancer and TGA. A few limitations of the present study should also be discussed. First, the identification of TGA in our study relied on hospital administrative records (i.e., inpatient and outpatient hospital visit), and as a result we were only able to identify TGA cases admitted over-night in a hospital or treated by a hospital-based specialist. Furthermore, since only >80% of the outpatient specialist visits are covered by the Patient Register, we likely had misclassified some TGA cases as TGA-free during follow-up. Nevertheless, the completeness of TGA ascertainment might not be a large concern as we observed a similar incidence rate of TGA among the cancer-free individuals (12 per 100,000 person-years) as previously reported (5–11 per 100,000 person-years [3–5]). If anything, such misclassification should be of larger concern for cancer-free individuals than cancer patients given the intensive medical surveillance among the latter, and therefore potentially leading to an artifact of the association between cancer and TGA. The null association observed in the present study alleviated such concern. On the contrary, the hospital-based TGA diagnosis did however have satisfactory specificity according to our validation study (93%). However, since the validation study was performed on the basis of two hospitals in the central part of Sweden, we were not able to assess the accuracy of TGA diagnosis from other parts of Sweden. Given the register-based nature of the present study, we had limited clinical information on other potential effect modifiers of the studied association, including cancer treatment. However, we did not find the association to vary clearly according to different time periods after cancer diagnosis or across different cancer types. Finally, in the present study we included only cancer patients diagnosed at age 30 and above since we found no TGA cases among cancer patients diagnosed at younger ages during the study period. Future studies are needed to specifically examine whether an association of cancer diagnosis with TGA exists among people younger than 30 years.

In conclusion, based on a large cohort study of more than five million Swedes, we did not find a positive association between a cancer diagnosis and the risk of TGA.
